# Sexual and genotypic variation in terpene quantitative and qualitative profiles in the dioecious shrub *Baccharis salicifolia*

**DOI:** 10.1038/s41598-019-51291-w

**Published:** 2019-10-10

**Authors:** Xoaquín Moreira, Luis Abdala-Roberts, Colleen S. Nell, Carla Vázquez-González, Jessica D. Pratt, Ken Keefover-Ring, Kailen A. Mooney

**Affiliations:** 1 Misión Biológica de Galicia (MBG-CSIC), Apdo. 28, 36080 Pontevedra, Galicia Spain; 2 Department of Tropical Ecology, Autonomous University of Yucatan, Apartado Postal 4-116, Itzimna. 97000, Merida, Yucatan Mexico; 3 Department of Biological Sciences, The George Washington University, Washington, DC 20052 USA; 4 University of California, Department of Ecology and Evolutionary Biology, Irvine, California 92697 USA; 5 University of Wisconsin-Madison, Departments of Botany and Geography, Madison, WI 53705 USA

**Keywords:** Chemical ecology, Ecological genetics

## Abstract

Terpenoids are secondary metabolites produced in most plant tissues and are often considered toxic or repellent to plant enemies. Previous work has typically reported on intra-specific variation in terpene profiles, but the effects of plant sex, an important axis of genetic variation, have been less studied for chemical defences in general, and terpenes in particular. In a prior study, we found strong genetic variation (but not sexual dimorphism) in terpene amounts in leaves of the dioecious shrub *Baccharis salicifolia*. Here we build on these findings and provide a more in-depth analysis of terpene chemistry on these same plants from an experiment consisting of a common garden with male (N = 19) and female (N = 20) genotypes sourced from a single population. Our goal in the present study was to investigate quantitative and qualitative differences in terpene profiles associated with plant sex and genotypic variation. For this, we quantified leaf mono- and sesquiterpene amount, richness, and diversity (quantitative profile), as well as the composition of compounds (qualitative profile). We found no evidence of sexual dimorphism in monoterpene or sesquiterpene profiles. We did, however, find significant genotypic variation in amount, diversity, and composition of monoterpenes, but no effects on sesquiterpenes. These findings indicated that genotypic variation in terpene profiles largely surpassed variation due to sexual dimorphism for the studied population of this species.

## Introduction

Terpenoids encompass a group of secondary metabolites which are often produced in high amounts in most plant tissues^1^. They are typically classified based on the number of carbon atoms of a molecule, namely: monoterpenes (C_10_), sesquiterpenes (C_15_), diterpenes (C_20_), triterpenes (C_30_), and tetraterpenes (C_40_). Due to their low molecular weight, mono- and sesquiterpenes are highly volatile components found in scents and fragrances emitted by aromatic plants^1^. Many of these volatile compounds are considered as toxic or repellent to herbivores and pathogens^1,2^. In addition, they can also play multiple other roles in plant-insect interactions. These include attraction of predatory arthropods and parasitoids^3–5^, attraction of insect pollinators and seed dispersers^6–8^, insect-insect interactions such as co-factors for bark beetle aggregation pheromones^9^, plant-to-plant communication as warning signals to neighbouring plants of herbivore presence^10,11^, and plant protection against abiotic stresses (e.g. drought or elevated temperatures^12,13^).

A number of studies have reported substantial variation both within and among populations in terpene quantitative profiles, mainly for shrubs and trees^14–20^. As for other plant defensive traits involved in herbivore resistance, studies have assessed broad-sense genetic variation in terpene levels (i.e. “genotypic” effects in ecological studies or Quantitative Trait Loci^14,16,21,22^) and in some cases addressed specific genes or groups of genes that code for focal compounds (e.g. candidate genes^23^). Plant sex is an ecologically important form of genetic variation in dioecious plants^24^. Dioecy is frequently characterized by the presence of sexual dimorphism in various traits^25^, which includes defensive traits associated with resistance to herbivores^26^. Female plants are expected to invest more resources into reproduction than males, such that allocation trade-offs are expected to lead to decreased growth and in turn higher investment in defensive traits relative to males^27,28^. Despite mounting evidence of sexual dimorphism in traits associated with resistance to herbivory, including plant physical defences (e.g. spines^29^, leaf toughness^30^) and secondary chemistry (e.g. phenolic compounds^28,30^ or coumarins^31^), few studies have tested for effects of plant sex on terpenes^32,33^. In addition, although there are a number of studies measuring the effects of plant sex on quantitative variation in chemical defences, including terpenes^32,33^, fewer have tested for effects on compositional variation or assessed the effects of plant sex relative to other sources of genetic variation. As a result, the degree of quantitative or qualitative variation in chemical defences between sexes (i.e. effects of sex on population genetic structure in defences) and the contribution of plant sex to variation in defences relative to total genetic variation associated with defences or that from other sources of ecologically important genetic variation are unknown. Disentangling these different sources of variation and their degree of control over plant phenotypes is important to gain a mechanistic understanding of genetic variation underlying plant chemical defences.

*Baccharis salicifolia* (Ruiz & Pav.) Pers. (Asteraceae) is a woody shrub for which sex is likely genetically determined^34^. Our previous work with this plant species showed genetic variation in several traits related to growth and reproduction (e.g. flower number, relative growth rate^33^), in the emission of plant volatile organic compounds^35^, and in arthropod abundance and composition^24,33,36^, as well as sexual dimorphism in several plant traits (more flowers and higher growth rate for females compared to males) and arthropod community composition^33^. Likewise, in a recent study we also found substantial genetic variation (but not sexual dimorphism) in leaf terpene amount for this species^33^. Here we build on these recent findings and provide a more in-depth analysis of terpene variation for these same plants from an experiment consisting of a common garden with male (N = 19) and female (N = 20) genotypes sourced from a single population of *B. salicifolia*^33^. Specifically, we quantify leaf mono- and sesquiterpene amount, richness, and diversity (i.e. quantitative profile) as well as compound composition (i.e. qualitative profile). By replicating multiple genotypes within each sex, we are able to compare the effects of plant sex vs. those due to additional genotypic variation, and in doing so provide a unique assessment of multiple sources of genetic variation not only in quantitative but also qualitative terpene expression.

## Results

We detected a total of 56 terpenoid compounds in *B. salicifolia* leaf tissue, of which 25 were monoterpenes and 31 were sesquiterpenes. Of this total, 46 were positively identified (Table 1). On average, terpenoid compounds comprised 13.5 ± 1.1% SE of leaf fresh weight (range: 0.44–91.1%) with sesquiterpenes representing 81.2% of this total. The five compounds found at highest amounts, together accounting for an average of 59.72 ± 1.4% SE (range: 4.0–25.5%) of total terpene amount, were the monoterpenes limonene and (E)-β-ocimene, and the sesquiterpenes α-bisabolol, cuprenen-1-ol (4-), and chromolaenin (Table 1).

**Table 1:** Amount, estimated as normalized peak area per fresh weight, of monoterpenes and sesquiterpenes in leaves of *Baccharis salicifolia* female and male plants belonging to 39 genotypes (N = 19 males and N = 20 females).

	KRI	Female	Male
**Monoterpenes**			
α-Pinene	933	2.293 ± 0.971	3.468 ± 0.993
Camphene	956	3.141 ± 1.061	3.168 ± 1.076
Sabinene	972	3.768 ± 1.005	3.673 ± 1.019
β-Pinene	983	11.002 ± 1.282	9.220 ± 1.299
α-Phellandrene	1008	9.903 ± 1.656	10.044 ± 1.679
Limonene	1028	44.508 ± 9.759	50.414 ± 9.896
(*E*)-β-Ocimene	1052	90.844 ± 13.687	111.330 ± 13.879
γ-Terpinene	1066	38.684 ± 9.332	36.515 ± 9.463
Linalool	1094	3.098 ± 0.839	3.244 ± 0.851
1,3,8-*p*-Menthatriene	1108	6.878 ± 0.991	4.813 ± 1.005
*trans*-Pinocarveol	1134	3.536 ± 1.405	4.478 ± 1.425
*trans*-Verbenol	1147	3.595 ± 2.245	8.729 ± 2.276
Pinocarvone	1163	3.655 ± 1.486	2.224 ± 1.507
Terpinen-4-ol	1176	2.025 ± 1.171	2.656 ± 1.188
α-Terpineol	1184	1.888 ± 0.914	2.570 ± 0.927
Myrtenol	1197	3.634 ± 1.546	3.513 ± 1.568
β-Cyclocitral	1225	2.383 ± 1.819	5.428 ± 1.844
Carvone	1244	2.406 ± 1.268	3.556 ± 1.286
Geranial	1258	2.006 ± 1.382	3.796 ± 1.402
Perilla aldehyde	1271	3.103 ± 1.479	2.432 ± 1.499
*p*-Menth-1-en-7-al	1280	0.857 ± 0.593	1.374 ± 0.601
Unknown 1	1287	0.626 ± 0.364	0.862 ± 0.369
Perilla alcohol	1294	1.538 ± 0.827	2.106 ± 0.839
Sabinyl acetate	1305	1.597 ± 0.576	0.928 ± 0.584
2E-,4E-Decadienal	1316	1.549 ± 1.886	5.050 ± 1.912
**Sesquiterpenes**			
Myrtenyl acetate	1330	5.226 ± 1.525	1.886 ± 1.546
Eugenol	1362	3.172 ± 0.686	1.971 ± 0.696
δ-Elemene	1342	1.256 ± 0.695	2.593 ± 0.705
α-Cubebene	1349	8.160 ± 1.378	3.452 ± 1.397
α-Ylangene	1372	5.237 ± 1.670	5.704 ± 1.694
α-Copaene	1380	7.258 ± 1.595	6.783 ± 1.618
β-Caryophyllene	1418	4.840 ± 1916	6.731 ± 1.943
γ-Elemene	1441	3.544 ± 1.235	4.998 ± 1.252
α-Humulene	1455	17.807 ± 2.429	22.646 ± 2.463
γ-Muurolene	1477	32.787 ± 2.773	31.618 ± 2.812
α-Farnesene	1508	52.522 ± 3.811	46.090 ± 3.865
δ-Cadinene	1538	50.631 ± 4.697	44.831 ± 4.763
Elemol	1555	29.034 ± 2.946	26.115 ± 2.987
Ledol	1570	32.837 ± 4.375	30.868 ± 4.436
T-Cadinol	1610	9.841 ± 3.582	11.532 ± 3.632
γ-Eudesmol	1623	3.391 ± 0.804	2.942 ± 0.815
Cubenol	1648	20.526 ± 3.343	21.865 ± 3.390
α-Bisabolol	1675	190.420 ± 25.761	180.350 ± 26.123
4-Cuprenen-1-ol	1705	481.080 ± 80.704	396.310 ± 81.838
Chromolaenin	1731	237.290 ± 89.407	117.850 ± 90.664
Unknown 2	1753	11.432 ± 2.700	8.037 ± 2.738
γ-Curcumen-15-al	1776	17.011 ± 3.336	21.347 ± 3.383
Unknown 3	1795	57.399 ± 6.387	54.491 ± 6.476
Unknown 4	1819	55.391 ± 7.426	33.021 ± 7.530
Unknown 5	1855	6.083 ± 7.129	17.552 ± 7.229
Unknown 6	1866	12.679 ± 1.952	15.214 ± 1.979
Unknown 7	1885	11.566 ± 3.466	11.031 ± 3.515
Unknown 8	1908	15.010 ± 3.325	10.471 ± 3.372
Phytol	1936	31.192 ± 3.957	32.903 ± 4.012
Unknown 9	1967	2.679 ± 0.949	3.826 ± 0.962
Unknown 10	2000	4.979 ± 1.275	5.745 ± 1.293

Least-square means ± SE are shown. Compounds are following an ascending order of Kovats retention index (KRI).

### Sexual dimorphism and genotypic variation in terpene quantitative profile

**Figure 1:**
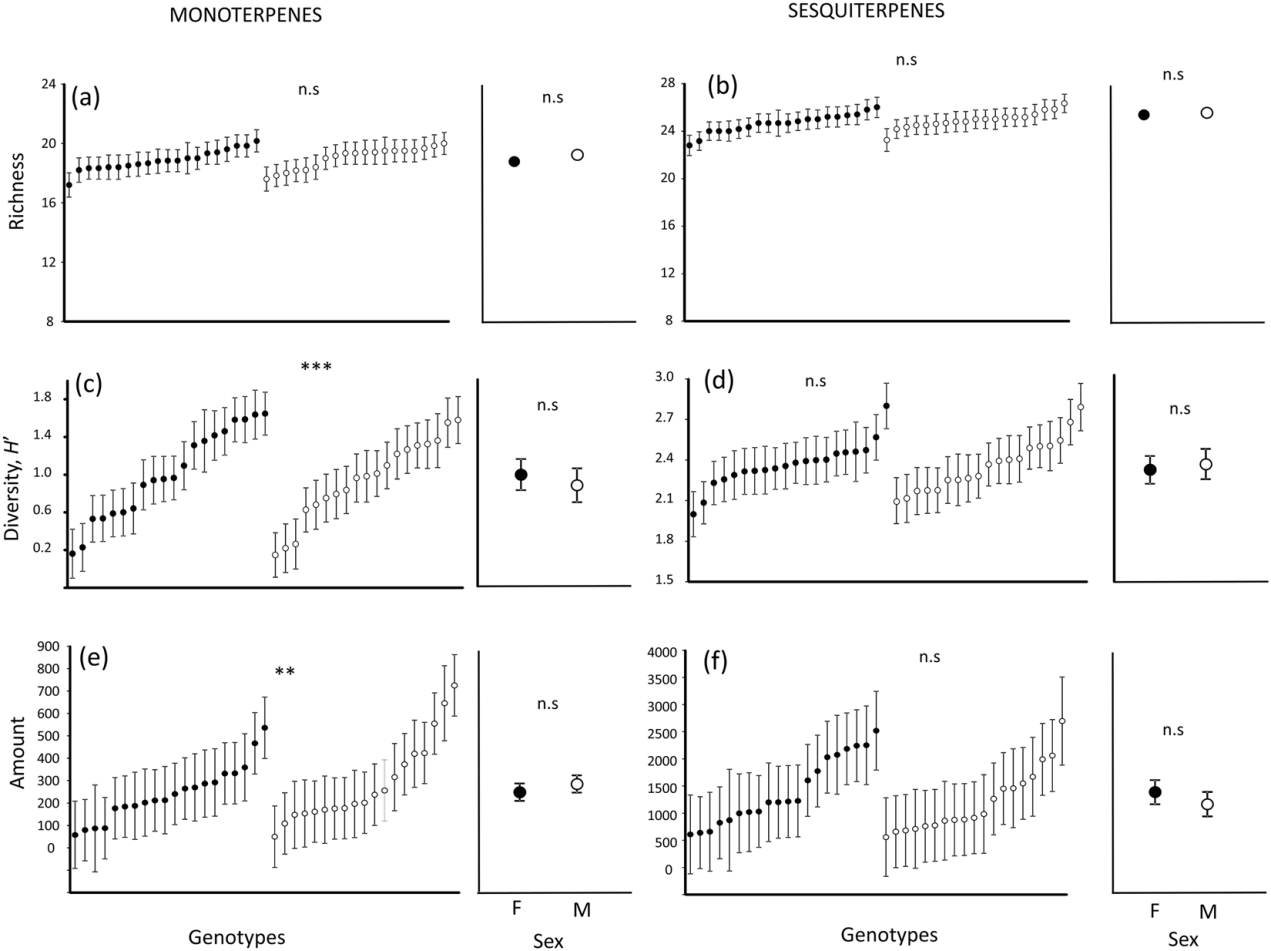
Genotypic variation and sexual dimorphism in terpene chemistry. Genotypic variation and sexual dimorphism in (**a**,**b**) richness (number of compounds), (**c**,**d**) diversity (H’; Shannon–Weiner index) and (**e**,**f**) amount (estimated as normalized peak area per fresh weight) of monoterpenes and sesquiterpenes in *Baccharis salicifolia* female (filled circles) and male (open circles) plants belonging to 39 genotypes (N = 19 males and N = 20 females). Circles are means ± s.e.m. Asterisks indicate significant differences between plant genotypes or sexes at *P* < 0.01 (**). n.s. = non-significant.

We found no detectable effect of plant sex on richness, diversity or amount of either mono- or sesquiterpenes (Table 2, Fig. 1a–f). We did, however, find significant genotypic variation in diversity and amount of monoterpenes in *B. salicifolia* (Table 2). Specifically, there was up to 11.7-fold and 14.6-fold variation in diversity (measured as the Shannon–Weiner index, H’; range: 0.14 ± 0.24 to 1.64 ± 0.23, Fig. 1c) and amount (range: 49.69 ± 136.98 to 725.11 ± 136.98 normalized peak area per fresh weight, Fig. 1e) of monoterpenes between plant genotypes. We found no evidence of spatial autocorrelation (e.g. clustering) of monoterpene diversity and amount, but rather these two variables were homogeneously distributed throughout the study area (Fig. S1 in the Supplementary Material). We did not find genotypic variation in richness of monoterpenes (Table 2, Fig. 1a), or in richness, diversity, or amount of sesquiterpenes (Table 2, Fig. 1b,d,f).

**Table 2:** Summary of results from linear mixed models testing for the effect of plant sex and plant genotype nested within sex in richness, diversity (H’) and amount of monoterpenes and sesquiterpenes in *Baccharis salicifolia* plants belonging to 39 genotypes (N = 19 males and N = 20 females).

Variable	Plant sex		Plant genotype	
	F_1,175_	*P*	F_37,175_	*P*
Richness – Monoterpenes	0.51	0.477	0.85	0.719
Richness – Sesquiterpenes	1.53	0.218	0.79	0.801
H’ – Monoterpenes	0.53	0.468	4.75	<**0.001**
H’ – Sesquiterpenes	0.01	0.978	1.24	0.180
Amount – Monoterpenes	0.05	0.832	1.95	**0.002**
Amount – Sesquiterpenes	0.04	0.835	0.72	0.885

We also included the block as a random factor. F-values with degrees of freedom (numerator, denominator) and associated significance levels (*P*-values) are shown. Significant *P*-values (*P* < 0.05) are highlighted in bold face.

### Sexual dimorphism and genotypic variation in terpene qualitative profile

**Figure 2:**
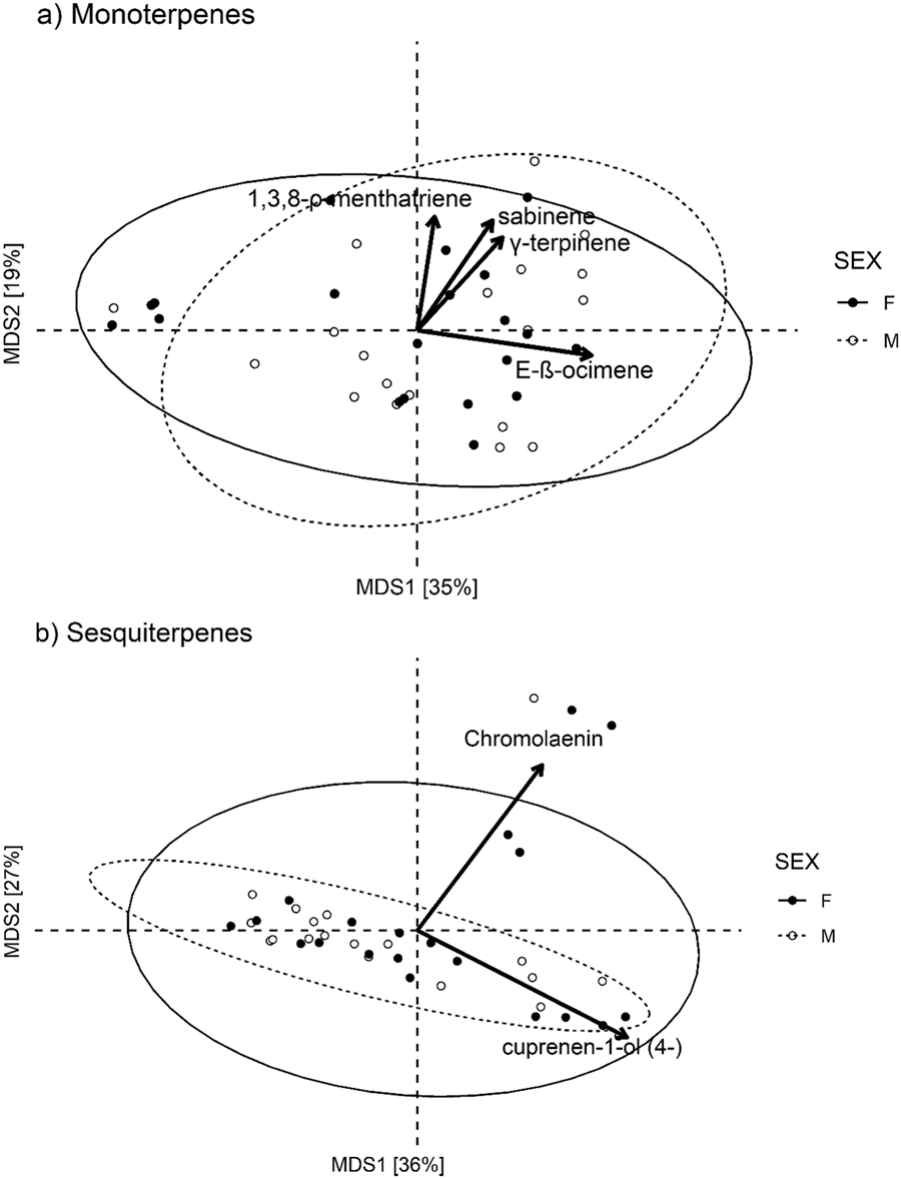
Unconstrained ordinations of sexual dimorphism in terpene composition. Unconstrained ordinations of sexual dimorphism in (**a**) monoterpene and (**b**) sesquiterpene composition. Biplot arrows show associated linear trends with terpenes, scaled to reflect relative magnitude of effects based on R^2^ values (R^2^ > 0.60, *P* < 0.001). The sex ordination displays male and female centroids and 95% ellipses, as well as the means for each genotype. For monoterpenes (panel a), the PERMANOVA indicates that 0.02% of monoterpene composition variation is explained by sex. Overall, the first two axes of ordination accounted for 54% of the genotypic variation in monoterpene composition (35% and 19% respectively). For sesquiterpenes (panel b), the PERMANOVA indicates that 0.02% of sesquiterpene composition variation is explained by sex. Overall, the first two axes of ordination accounted for 63% of the genotypic variation in sesquiterpene composition (36% and 27% respectively). Female (N = 20) and male (N = 19) genotypes are depicted as closed and open circles, respectively.

**Figure 3:**
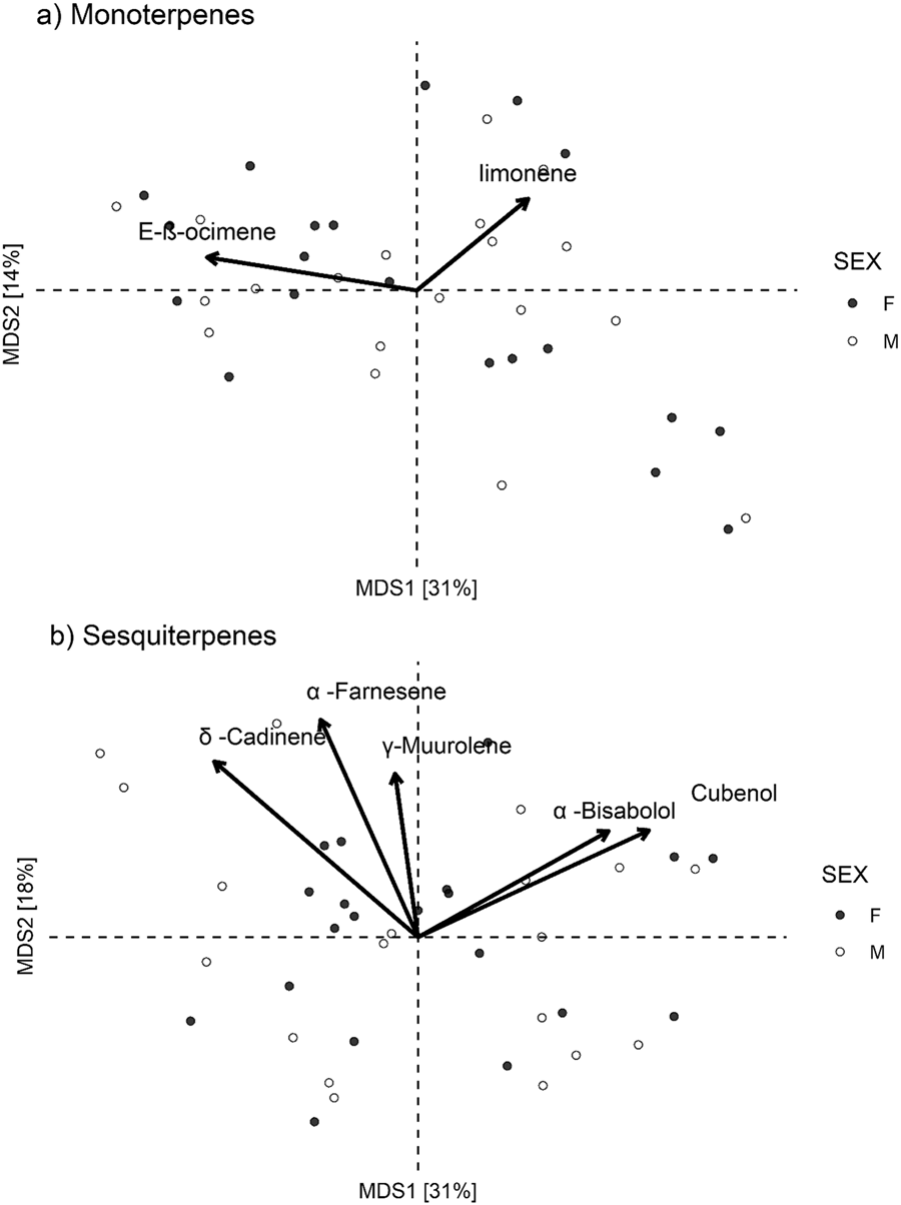
Unconstrained ordinations of genotypic variation in terpene composition. Unconstrained ordinations of genotypic variation in (**a**) monoterpene and (**b**) sesquiterpene composition. Biplot arrows show associated linear trends with terpenes, scaled to reflect relative magnitude of effects based on R^2^ values (R^2^ > 0.35, *P* < 0.001). This genotypic ordination displays genotypic centroids while controlling for sexual dimorphism. For monoterpenes (panel a), the PERMANOVA (controlling for the effects of sex) indicates that 23% and 25% of monoterpene composition variation is explained by male and female genotypic variation, respectively. Overall, the first two axes of ordination accounted for 45% of the genotypic variation in monoterpene composition (31% and 14% respectively). For sesquiterpenes (panel b), the PERMANOVA (controlling for the effects of sex) indicates that 20% and 16% of sesquiterpene composition variation is explained by male and female genotypic variation, respectively. Overall, the first two axes of ordination accounted for 49% of the genotypic variation in sesquiterpene composition (31% and 18% respectively). Female (N = 20) and male (N = 19) genotypes are depicted as closed and open circles, respectively.

The PERMANOVA test of sexual dimorphism in qualitative profiles indicated that neither monoterpene (pseudo-F = 0.62, df = 1, *P* = 0.685, Fig. 2a) nor sesquiterpene (pseudo-F = 0.65, df = 1, *P* = 0.628, Fig. 2b) composition differed across plant sexes. On the other hand, the PERMANOVA for genotypic variation indicated that monoterpene (pseudo-F = 1.47, df = 38, *P* < 0.001, Fig. 3a), but not sesquiterpene (pseudo-F = 1.02, df = 38, *P* = 0.354, Fig. 3b), composition varied across genotypes. The PERMANOVA constrained by plant sex indicated that 23% and 25% of the variation in monoterpene composition was explained by male and female genotypic variation, respectively. The first two axes of the ordination together accounted for 45% of the genotypic variation in monoterpene composition (31% and 14% respectively, Fig. 3a). Genotypic variation in monoterpene composition was primarily associated with variation in the relative amount of limonene (R^2^ = 0.41, *P* < 0.001) and (*E*)-β-ocimene (R^2^ = 0.44, *P* < 0.001) (Fig. 3a).

## Discussion

Our findings indicated that sesquiterpenes were overall more abundant than monoterpenes in the analyzed *B. salicifolia* leaf samples, but only the latter exhibited significant genotypic variation in quantitative and qualitative profiles and we did not find sexual dimorphism in either terpene group. There was significant genotypic variation in monoterpene amount, diversity and composition (but not richness), with a few noticeable compounds dominating the samples (e.g. limonene and (E)-β-ocimene). In addition, we also found no sexual dimorphism in monoterpene or sesquiterpene amount, richness, diversity and composition. It therefore appears from our comprehensive analyses that plant sex is not a relevant axis of genetic variation in terpene quantitative and qualitative profiles in *B. salicifolia*. This, however, does not preclude the presence of sexual dimorphism in other chemical (e.g. diterpenes, triterpenes, phenolic compounds) or physical (e.g. toughness) defensive traits of potential importance to plant-herbivore or other types of interactions in this species.

Our results indicated no evidence of sexual dimorphism in terpene quantitative or qualitative measures. Theory predicts that female plants should invest more energy in reproduction and defence and less in growth relative to male plants^27,28^. Our findings do not support this prediction and add to a growing number of studies reporting inconsistent patterns with either male plants being more highly defended or no difference between sexes (reviewed by Avila-Sakar & Romanow^37^). For example, Stark and Martz^32^ found no sexual dimorphism in terpene concentration in shoots of *Juniperus communis*. Similarly, our previous work based on the same *B. salicifolia* experimental plants used here indicated weak or non-detectable sexual dimorphism in plant traits associated with growth and reproduction as well as in arthropod community structure associated with this plant^33^. Although speculative, the observed lack of sexual variation in terpene chemistry for *B. salicifolia* could have implications for herbivore preference or performance. For example, in another previous study we found that male *B. salicifolia* plants had higher abundances of the generalist aphid *Aphis gossypii*, whereas plant sexes did not differ in abundance of the specialist aphid *Uroleucon macolai*^24^. If terpene chemistry matters for herbivore preference or performance, taken together, results from that study and our current work suggest that higher *A. gossypii* numbers on male plants respond to plant chemical (or physical) traits other than terpenes that potentially do show differences between plant sexes (e.g. nutrients, architecture, etc.). In the case of *U. macolai*, this aphid could be responsive to these compounds such that a lack of sexual dimorphism in terpene chemistry would preclude concomitant variation in this aphid’s abundance, or, alternatively, this aphid could also be affected but overcomes plant sex differences in other defensive traits.

Our results showed that monoterpene diversity and amount exhibited variation among *B. salicifolia* genotypes. Previous work has similarly reported variation in the amount of leaf monoterpenes both between and within populations for a number of shrub and tree species^14–19,38^. Several of these studies have found that the amount of these compounds is associated with resistance to insect herbivory^1,39–41^, suggesting a defensive role in plant-herbivore interactions. On the other hand, studies reporting on intraspecific genetic variation in monoterpene diversity are much more limited. One exception is a recent study of ours where we also found that monoterpene diversity exhibited significant variation among populations of *Artemisia californica* distributed along a latitudinal gradient in California^19,42^. Genetic variation in terpene diversity could also be potentially important as a number of studies have shown that greater chemical diversity is associated with increased resistance against herbivores^43,44^, and may buffer populations against other sources of biotic stress (e.g. pathogens) or abiotic (e.g. temperature) stress. Although there are a number of studies reporting on phenotypic variation in secondary chemistry in the genus *Baccharis* (including *B. salicifolia*), these have not involved explicit assessments of sources of genetic variation in chemical traits^45–47^. In this sense, our results provide information on genotypic variation in the quantitative terpene profile for this species. The fact that we detected significant variation in these quantitative traits within a single population warrants future work assessing variation across populations and its potential biotic or abiotic correlates, as well as experimental studies investigating the influence of monoterpene amount and diversity on herbivore resistance.

We also found significant variation in monoterpene composition among *B. salicifolia* genotypes. Similarly, a recent work by our group showed significant variation in monoterpene composition across populations of *A. californica*^19^. In addition, Thompson *et al*.^48^ similarly reported significant variation in monoterpene composition across populations of *Thymus vulgaris*. It should be noted, however, that despite observing genotypic variation in monoterpene composition, we did not find evidence of distinct ‘chemotypes’ within the studied population as reported for other species, primarily of Mediterranean climate origin^49,50^. Genotypic variation in monoterpene composition in *B. salicifolia* was primarily associated with changes in the relative amounts of two major compounds (limonene and (*E*)-β-ocimene), which did not separate into distinct genotypic groups but rather exhibited a range of variation in relative abundances across genotypes. Previous studies have reported that these two compounds may act as repellents or toxins to herbivores in woody species^14,44,51–53^. In particular, we previously found that the emission of both limonene and E-β-ocimene drastically increased after aphid herbivory^54^ so unaccounted differences in herbivory on our experimental plants could have influenced (via induced responses) observed patterns of genotypic variation in constitutive terpene profiles.

We found no detectable genotypic variation in any of the quantitative or qualitative measures of variation in sesquiterpenes. With respect to quantitative measures, a number of studies have shown significant intra-specific variation in sesquiterpene amount^39,55,56^, but the magnitude of variation in these compounds appears to be lower compared to monoterpenes^14,19,57^. For example, Sampedro *et al*.^14^ reported lower genotypic variation for sesquiterpene amount than for monoterpenes in young trees of *Pinus pinaster* in north-western Spain. Similarly, previous work of ours indicated that monoterpene but not sesquiterpene richness and diversity varied significantly across populations of *A. californica*^19^. In addition, and consistent with quantitative profiles, we found no evidence of genotypic variation in the composition of sesquiterpenes in *B. salicifolia*. To our knowledge, only two previous studies have tested for intra-specific variation in sesquiterpene composition and, in contrast to our study, both reported significant variation among populations^19,57^. In particular, Pratt *et al*.^19^ found that sesquiterpene composition in *A. californica* significantly varied among populations distributed along the Californian coast, whereas Moniodis *et al*.^57^ found that sesquiterpene composition in leaves of *Santalum spicatum* trees significantly varied across populations distributed in arid regions of West Australia. As compared to our study of within-population variation, both of these studies assessed variation among-population, and this distinction may underlie the contrasting results.

Our findings provide an assessment of quantitative and qualitative variation in terpene profiles in *B. salicifolia* and its underlying genetic sources. Additional work involving multiple populations of *B. salicifolia*, as well as measurements of terpenes in other plant tissues (e.g. flowers), are necessary to reach stronger conclusions about sex variation in terpene chemistry as well as assess the independent effects of different sources (e.g. sexual vs. non-sexual) of genotypic variation on terpene expression. For example, there may be genes associated with variation in terpene profiles that are linked to genes that determine sex^25^, thus appearing spuriously associated. Conducting controlled crosses with different populations to produce segregating progeny would allow for a test of sex by genotype interactions to assess the linkage between sex-related and unrelated genetic variation. In addition, further work involving population variation, e.g. along ecological gradients in herbivory or abiotic variables, would provide a useful next step for identifying relevant factors associated with genetic variation in defensive chemistry in this species. Additional work could involve experimental tests of the effects of such factors on terpene expression and its consequences for insect herbivores. As a whole, the present study points at the need of assessing the independent effects of different sources of genetically-based variation, including plant sex, concurrently shaping plant defensive traits to uncover the mechanistic basis of plant defensive phenotypes. Likewise, our findings also emphasize the importance of increasing the level of detail and comprehensiveness of analyses of chemical traits putatively associated with defences to fully describe complex chemical defensive phenotypes in plants as well as their role in herbivore resistance.

## Methods and Materials

### Study system

*Baccharis salicifolia* is a perennial, dioecious shrub widely distributed from the desert southwest of the United States and northern Mexico to South America^58,59^. It is typically found in riparian areas and mesic microhabitats in high-density monospecific stands, where multiple genotypes co-occur at small spatial scales^58,60^. In coastal southern California, *B. salicifolia* grows and flowers predominantly during the annual winter rains, but may also flower sporadically during the spring and fall. Notably, this species emits large amounts of volatile mono- and sesquiterpenes in all tissues (leaves, stems, flowers), and previous work suggests that these compounds confer protection against herbivores^35,54,61^ and abiotic stress (e.g. drought^47^).

This study provides a detailed analysis of terpene data from Nell *et al*.^33^. In that study, an experimental common garden was used to characterize sexual dimorphism and genetic variation in *B. salicifolia* traits and plant-associated arthropod communities. Monoterpene amount was shown to vary nearly 10-fold among 39 plant genotypes, while sesquiterpene amount did not vary significantly, and there was no sexual dimorphism in either compound class. In this study, we provide a detailed analysis of these data on chemical amount, richness, and diversity (i.e. quantitative profile) as well as compound composition (i.e. qualitative profile).

### Genotype selection, propagation and common garden

We used a source population of *B. salicifolia* occurring in 80 ha of habitat found within the University of California San Joaquin Marsh Reserve (33.66°N, 117.85°E; Orange County, CA, USA) that was also used in previous work of ours with this species^24,33,59,60,62^. In February 2008, we collected cuttings from 20 male and 20 female plants (i.e. genotypes hereafter). To maximize variation among genotypes, we collected cuttings from wild-grown plants that were separated by approximately 900 m. Cuttings were dipped in a 20% solution of Dip ‘N Grow Root Inducing Concentrate (Dip ‘N Grow Inc., Clackamas, OR), planted in perlite, and kept in a greenhouse for six weeks. We then planted all cuttings in 1 L pots of soil (equal parts silica sand, redwood compost, peat moss, and pumice) where they continued to grow for two months. One male genotype did not propagate successfully and was therefore eliminated from the study.

### Common garden

In May 2008, we established a common garden of *B. salicifolia* adjacent to the Marsh Reserve. We planted 39 genotypes and replicated each genotype 8–13 times (mean 11.5 ± 0.2; total N = 459 plants). We randomly distributed plants throughout the common garden in rows and columns with 1 m spacing between them (Fig. S2 in the Supplementary Material), and we divided the garden into 12 spatial blocks to account for soil heterogeneity. We watered plants with city water using drip irrigation emitters twice a week.

### Terpene analyses

In November 2011, we collected two fully expanded (undamaged) sun-exposed leaves from half of the replicates (5–6) for each genotype (N = 215 plants; Fig. S2). For terpene extraction, we immediately weighted the collected leaves and placed them in small pieces into 2 ml n-hexane (99.9% purity), sonicated them for 10 min and allowed them to soak at room temperature for seven days^19^. We then poured off the extracts and stored them at −80 °C. For the terpene analysis, we added 10 μL of an internal standard solution (0.13 μL mL^−1^ *m*-xylene in *n*-hexane) to 90 μL of each sample extract. We injected the samples (4 μL) onto a gas chromatograph (GC, ThermoFinnegan TraceMS+, Waltham, MA, USA) with a mass spectrometer (MS) detector that was fitted with a 30 m × 0.25 mm × 0.25 μ film thickness DB-5 fused silica column. The GC was operated in splitless mode with helium as the carrier gas (flow rate 1 mL min^−1^). The GC oven temperature program was: 1 min hold at 50 °C, 5 °C min^−1^ ramp to 180 °C, 20 °C min^−1^ ramp to 290 °C, and 1 min hold at 290 °C. The MS was operated in electron ionization mode at 70.0 eV and we collected data between 50–650 m/z. We identified mono- and sesquiterpenes using a NIST Mass Spectral Library and comparing their Kováts indices (Table 1), calculated relative to the retention times of a series of n-alkanes (C_8_-C_20_, Sigma-Aldrich, Merck KGaA, Darmstadt, Germany) analysed under the same chromatographic conditions, with those reported in the literature^46,63^. It is important to note that, although our Kováts indices matched well with those previously reported^45,63^, terpene compounds should be considered as ‘putative’ until confirmation with standards. For each plant, we estimated the amount of mono- and sesquiterpenes by using normalized peak areas per fresh weight. The normalized peak area per fresh weight of each compound was obtained by dividing their integrated peak area by the integrated peak area of the internal standard and then dividing this value by the leaf fresh weight. To assess the relative abundance of terpenes across plant genotypes and sexes, we also calculated mono- and sesquiterpene diversity for each plant using the Shannon–Weiner index: H’ = −Σ(P_i_ log[P_i_]), where P_i_ is the relative amount of a given terpene divided by the total terpenes in each plant. Finally, we also recorded the total number of mono- and sesquiterpene compounds (i.e. richness).

### Statitical analyses

#### Sexual dimorphism and genotypic variation in terpene quantitative profiles

We ran linear mixed models including plant sex and plant genotype nested within sex as fixed factors to test for sexual dimorphism and genotypic variation in richness, diversity and amount of monoterpenes and sesquiterpenes (i.e. quantitative profile). We also included block as a random factor. We ran all analyses with PROC MIXED in SAS 9.4 (SAS Institute, Cary, NC)^64^. We log-transformed all variables to achieve normality of residuals, and reported least square means ± SE in the original (untransformed) scale as descriptive statistics.

#### Sexual dimorphism and genotypic variation in qualitative terpene profiles

We tested for the effect of plant genotype on mono- and sesquiterpene composition (i.e. qualitative profile) separately using data on the relative amount of individual compounds for each type of terpene. We used a permutational multivariate analyses of variance (PERMANOVA)^65^ including plant genotype as a fixed factor, constrained by plant sex to control for any effects of sexual dimorphism. A PERMANOVA is analogous to an ANOVA, but partitions similarity matrices between treatments and uses permutation tests with pseudo F-ratios. The PERMANOVA was based on 10,000 permutations using the ‘vegan’ package^66^ in R software^67^. To visualize the results of this analysis, we used pairwise Bray-Curtis dissimilarities as input to a principal coordinates analysis. The result of this analysis was then visualized in two dimensions, where each point reflected the genotype centroid. We selected influential terpenes based upon R^2^ > 0.35 (*P* < 0.001) for associations with the first two ordination axes (using ‘envfit’ in vegan) and displayed using biplot arrows with length scaled to R^2^ values.

We used the same procedures described previously using PERMANOVA to test for sexual dimorphism on terpene composition (qualitative profile) using genotype least square means. We visualized sexual dimorphism (with ordination) in terpene composition with the two sex centroids as well as the mean values for each male and female genotype displayed on the ordination plot. We selected influential terpenes based upon R^2^ > 0.60 (*P* < 0.001) for associations with the first two ordination axes.

## Supplementary information

Supplementary Material

## References

[1] Pichersky E, Raguso RA (2018). Why do plants produce so many terpenoid compounds?. New Phytologist.

[2] Seybold SJ, Huber DPW, Lee JC, Graves AD, Bohlmann J (2006). Pine monoterpenes and pine bark beetles: a marriage of convenience for defense and chemical communication. Phytochemistry Reviews.

[3] Dicke M, Baldwin IT (2010). The evolutionary context for herbivore-induced plant volatiles: beyond the “cry-for-help”. Trends in Plant Science.

[4] Turlings TCJ, Tumlinson JH, Lewis WJ (1990). Explotation of herbivore-induced plant odors by host-seeking parasitic wasps. Science.

[5] De Moraes CM, Lewis WJ, Paré PW, Alborn HT, Tumlinson JH (1998). Herbivore-infested plants selectively attract parasitoids. Nature.

[6] Dudareva N, Pichersky E (2000). Biochemical and molecular genetic aspects of floral scents. Plant Physiology.

[7] Reinhard J, Srivivasan MV, Zhang S (2004). Scent-triggered navigation in honeybees. Nature.

[8] Raguso RA (2008). Wake up and smell the roses: the ecology and evolution of floral scent. Annual Review of Ecology, Evolution, and Systematics.

[9] Erbilgin N, Powell JS, Raffa KF (2003). Effect of varying monoterpene concentrations on the response of *Ips pini* (Coleoptera: Scolytidae) to its aggregation pheromone: implications for pest management and ecology of bark beetles. Agricultural and Forest Entomology.

[10] Heil M, Karban R (2010). Explaining the evolution of plant communication by airborne signals. Trends in Ecology and Evolution.

[11] Karban, R. *Plant sensing and communication*. (The University of Chicago Press, 2015).

[12] Holopainen JK, Gershenzon J (2010). Multiple stress factors and the emission of plant VOCs. Trends in Plant Science.

[13] Peñuelas J, Llusià J (2002). Linking photorespiration, monoterpenes and thermotolerance in *Quercus*. New Phytologist.

[14] Sampedro L, Moreira X, Llusia J, Peñuelas J, Zas R (2010). Genetics, phosphorus availability and herbivore-derived induction as sources of phenotypic variation of leaf volatile terpenes in a pine species. Journal of Experimental Botany.

[15] Fady B, Arbez M, Marpeau A (1992). Geographic variability of terpene composition in Abies cephalonica Loudon and Abies species around the Aegean: hypotheses for their possible phylogeny from the Miocene. Trees.

[16] O’Reilly-Wapstra JM, Iason GR, Thoss V (2006). The role of genetic and chemical variation of *Pinus sylvestris* seedlings in influencing slug herbivory. Oecologia.

[17] Trapp S, Croteau R (2001). Defensive resin biosynthesis in conifers. Annual Review of Plant Physiology and Plant Molecular Biology.

[18] White, E. E. & Nilsson, J. E. Foliar terpene heritability in Pinus contorta. *Silvae genetica* **33** (1984).

[19] Pratt JD, Keefover-Ring K, Liu L, Mooney KA (2014). Genetically-based latitudinal variation in *Artemisia californica* secondary chemistry. Oikos.

[20] Huang M (2010). Variation of herbivore-induced volatile terpenes among Arabidopsis ecotypes depends on allelic differences and subcellular targeting of two terpene synthases, TPS02 and TPS03. Plant Physiology.

[21] Henery ML, Moran GF, Wallis IR, Foley WJ (2007). Identification of quantitative trait loci influencing foliar concentrations of terpenes and formylated phloroglucinol compounds in *Eucalyptus nitens*. New Phytologist.

[22] O’Reilly-Wapstra JM (2011). Quantitative trait loci for foliar terpenes in a global eucalypt species. Tree Genetics & Genomes.

[23] Chen F, Tholl D, Bohlmann J, Pichersky E (2011). The family of terpene synthases in plants: a mid-size family of genes for specialized metabolism that is highly diversified throughout the kingdom. The Plant Journal.

[24] Abdala-Roberts L (2016). Multi-trophic consequences of plant genetic variation in sex and growth. Ecology.

[25] Barrett SCH, Hough J (2013). Sexual dimorphism in flowering plants. Journal of Experimental Botany.

[26] Cornelissen T, Stiling P (2005). Sex-biased herbivory: A meta-analysis of the effects of gender on plant-herbivore interactions. Oikos.

[27] Eckhart, V. M. & Seger, J. In *Life history evolution in plants* (eds Vuorisalo, T. O. & Mutikainen, P. K.) 195–213 (Kluwer, 1999).

[28] Cepeda-Cornejo V, Dirzo R (2010). Sex-related differences in reproductive allocation, growth, defense and herbivory in three dioecious neotropical palms. PLoS ONE.

[29] Janczur MK (2014). Chemical and physical defense traits in two sexual forms of *Opuntia robusta* in Central Eastern Mexico. PLoS ONE.

[30] Jing SW, Coley PD (1990). Dioecy and herbivory: the effect of growth rate on plant defense in *Acer negundo*. Oikos.

[31] Alonso C, Pérez R, Nieto PM, Delgado J (2005). Gender dimorphism and altitudinal variation of secondary compounds in leaves of the gynodioecious shrub *Daphne laureola*. Journal of Chemical Ecology.

[32] Stark S, Martz F (2018). Gender dimorphism does not affect Secondary compound composition in *Juniperus communis* after shoot cutting in Northern boreal forests. Frontiers in Plant Science.

[33] Nell CS (2018). Relative effects of genetic variation sensu lato and sexual dimorphism on plant traits and associated arthropod communities. Oecologia.

[34] Ming R, Bendahmane A, Renner SS (2011). Sex chromosomes in land plants. Annual Review of Plant Biology.

[35] Moreira X, Nell CS, Meza-Lopez MM, Rasmann S, Mooney KA (2018). Specificity of plant-plant communication for Baccharis salicifolia sexes but not genotypes. Ecology.

[36] Mooney, K. A. & Singer, M. S. In *Ecology and Ev*oluti*on of Trait-Mediated Indirect Interactions: Linking Evolution, Community, and Ecosystem* (eds Ohgushi, T., Schmitz, O. & Holt, R. D.) (Cambridge University Press, 2012).

[37] Avila-Sakar G, Romanow CA (2012). Divergence in defence against herbivores between males and females of dioecious plant species. International Journal of Evolutionary Biology.

[38] Nerg A (1994). Seasonal and geographical variation of terpenes, resin acids and total phenolics in nursery grown seedlings of Scots pine (*Pinus sylvestris* L.). New Phytologist.

[39] López-Goldar X (2018). Inducibility of plant secondary metabolites in the stem predicts genetic variation in resistance against a key insect herbivore in maritime pine. Frontiers in Plant Science.

[40] Barnola LF, Hasegawa M, Cedeno A (1994). Mono- and sesquiterpene variation in Pinus caribaea needles and its relationship to Atta laevigata herbivory. Biochemical Systematics and Ecology.

[41] Chen Z, Kolb TE, Clancy KM (2002). The role of monoterpenes in resistance of Douglas fir to western spruce budworm defoliation. Journal of Chemical Ecology.

[42] Pratt JD, Mooney KA (2013). Clinal adaptation and adaptive plasticity in *Artemisia californica*: Implications for the response of a foundation species to predicted climate change. Global Change Biology.

[43] Iason GR (2005). Does chemical composition of individual Scots pine trees determine the biodiversity of their associated ground vegetation?. Ecology Letters.

[44] Thoss V, Byers JA (2006). Monoterpene chemodiversity of ponderosa pine in relation to herbivory and bark beetle colonization. Chemoecology.

[45] Loayza I (1995). Essential oils of *Baccharis salicifolia, B. latifolia* and *B. dracunculifolia*. Phytochemistry.

[46] He K, Montenegro G, Hoffmann JJ, Timmermann BN (1996). Diterpenoids from *Baccharis linearis*. Phytochemistry.

[47] Jakupovic J, Schuster A, Ganzer U, Bohlmann F, Boldt PE (1990). Sesqui- and diterpenes from *Baccharis* species. Phytochemistry.

[48] Thompson JD, Chalchat JC, Michet A, Linhart YB, Ehlers B (2003). Qualitative and quantitative variation in monoterpene co-occurrence and composition in the essential oil of *Thymus vulgaris* chemotypes. Journal of Chemical Ecology.

[49] Thompson JD (2013). Evolution of a genetic polymorphism with climate change in a Mediterranean landscape. Proceedings of the National Academy of Sciences.

[50] Karban R, Wetzel WC, Shiojiri K, Pezzola E, Blande JD (2016). Geographic dialects in volatile communication between sagebrush individuals. Ecology.

[51] Nordlander G (1990). Limonene inhibits attraction to α-pinene in the pine weevils *Hylobius abietis* and *H. pinastri*. Journal of Chemical Ecology.

[52] Mita E (2002). Seasonal variation of oleoresin terpenoids from *Pinus halepensis* and *Pinus pinea* and host selection of the scale insect *Marchalina hellenica* (Homoptera, Coccoidea, Margarodidae, Coelostonidiinae). Holzforschung.

[53] Iason GR, O’Reilly-Wapstra JM, Brewer MJ, Summers RW, Moore BD (2011). Do multiple herbivores maintain chemical diversity of Scots pine monoterpenes?. Philosophical Transactions of the Royal Society B.

[54] Moreira X, Nell CS, Katsanis A, Rasmann S, Mooney KA (2018). Herbivore specificity and the chemical basis of plant-plant communication in *Baccharis salicifolia* (Asteraceae). New Phytologist.

[55] Vourc’h G, Martin JL, Duncan P, Escarré J, Clausen TP (2001). Defensive adaptations of *Thuja plicata* to ungulate browsing: a comparative study between mainland and island populations. Oecologia.

[56] Blanch J-S (2012). Effects of phosphorus availability and genetic variation of leaf terpene contents and emission rates in *Pinus pinaster* seedlings susceptible and resistant to the pine weevil *Hylobius abietis*. Plant Biology.

[57] Moniodis J (2017). Sesquiterpene variation in West Australian sandalwood (*Santalum spicatum*). Molecules.

[58] Abad MJ, Bermejo P (2007). Baccharis (Compositae): a review update. Arkivoc 2007.

[59] Moreira X, Mooney KA (2013). Influence of plant genetic diversity on interactions between higher trophic levels. Biology Letters.

[60] Abdala-Roberts L, Pratt R, Pratt JD, Mooney K (2017). Traits underlying community consequences of plant intra-specific diversity. PLoS ONE.

[61] García M, Donadel OJ, Ardanaz CE, Tonn CE, Sosa ME (2005). Toxic and repellent effects of *Baccharis salicifolia* essential oil on *Tribolium castaneum*. Pest Management Science.

[62] Mooney KA, Pratt R, Singer MC (2012). The tri-trophic interactions hypothesis: Interactive effects of host plant quality, diet breadth and natural enemies on herbivores. PLoS ONE.

[63] Zunino MP, Novillo-Newton M, Maestri DM, Zygadlo JA (1997). Composition of the essential oil of *Baccharis crispa* Spreng. and *Baccharis salicifolia* Pers. grown in Córdoba (Argentina). Flavour and Fragrance Journal.

[64] Littell, R. C., Milliken, G. A., Stroup, W. W., Wolfinger, R. & Schabenberger, O. *SAS System for mixed models, second edition* (2006).

[65] Anderson MJ (2001). A new method for non-parametric multi-variate analysis of variance. Austral Ecology.

[66] Oksanen J (2016). Vegan: Community Ecology Package. R package version.

[67] R Core Team. R: A language and environment for statistical computing. R Foundation for Statistical Computing, Vienna, Austria. URL, http://www.R-project.org/ (2018).

