# Supplementary material for: Sexual and genotypic variation in terpene quantitative and qualitative profiles in the dioecious shrub Baccharis salicifolia
Source: Sci Rep. 2019 Oct 10;9:14655. doi: 10.1038/s41598-019-51291-w (PMC6787053; doi:10.1038/s41598-019-51291-w)
Supplement: Supplementary file 1 — Supplementary Material [file 41598_2019_51291_MOESM1_ESM.docx]

**Sexual and genotypic variation in terpene quantitative and qualitative profiles in the dioecious shrub** *Baccharis salicifolia*

Xoaquín Moreira^1*^, Luis Abdala-Roberts^2^, Colleen S. Nell^3^, Carla Vázquez-González^1^, Jessica D. Pratt^4^, Ken Keefover-Ring^5^, and Kailen A. Mooney^4*^

^1^Misión Biológica de Galicia (MBG-CSIC), Apdo. 28, 36080 Pontevedra, Galicia, Spain

^2^Department of Tropical Ecology, Autonomous University of Yucatan, Apartado Postal 4-116, Itzimna. 97000. Merida, Yucatan, Mexico

^3^Department of Biological Sciences, the George Washington University, Washington, DC 20052, USA

^4^Department of Ecology and Evolutionary Biology, University of California, Irvine, California, USA 92697

^5^ Departments of Botany and Geography, University of Wisconsin-Madison, Madison, WI, 53705, USA

**Figure S1.** Semivariograms of the residuals after adjusting for main effects in the model for monoterpene (a) diversity and (b) amount, showing the observed semivariance as a function of the distance separating plants. A reduction of the semivariance at short distances would indicate a patchy structure. The observed fairly flat semivariograms indicate random spatial variation for monoterpene diversity and amount.

**Figure S2.** Experimental design of the *Baccharis salicifolia* common garden established in May 2008. Each circle represents an individual plant; black circles indicate the subset of plants from Nell *et al.* (2018) used in the terpene analysis. The genotype of each plant is indicated by numbers 1-39, with male genotypes represented with open circles and female genotypes as filled circles.
